# Genetic diversity, infection prevalence, and possible transmission routes of *Bartonella* spp. in vampire bats

**DOI:** 10.1371/journal.pntd.0006786

**Published:** 2018-09-27

**Authors:** Daniel J. Becker, Laura M. Bergner, Alexandra B. Bentz, Richard J. Orton, Sonia Altizer, Daniel G. Streicker

**Affiliations:** 1 Odum School of Ecology, University of Georgia, Athens, Georgia, United States of America; 2 Center for the Ecology of Infectious Disease, University of Georgia, Athens, Georgia, United States of Ameirca; 3 Department of Microbiology and Immunology, Montana State University, Bozeman, Montana, United States of America; 4 Institute of Biodiversity, Animal Health and Comparative Medicine, University of Glasgow, Glasgow, United Kingdom; 5 Department of Poultry Science, University of Georgia, Athens, Georgia, United States of America; 6 Department of Biology, Indiana University, Bloomington, Indiana, United States of America; 7 MRC–University of Glasgow Centre for Virus Research, Glasgow, United Kingdom; Instituto de Pesquisas Veterinarias Desiderio Finamor, BRAZIL

## Abstract

*Bartonella* spp. are globally distributed bacteria that cause endocarditis in humans and domestic animals. Recent work has suggested bats as zoonotic reservoirs of some human *Bartonella* infections; however, the ecological and spatiotemporal patterns of infection in bats remain largely unknown. Here we studied the genetic diversity, prevalence of infection across seasons and years, individual risk factors, and possible transmission routes of *Bartonella* in populations of common vampire bats (*Desmodus rotundus*) in Peru and Belize, for which high infection prevalence has previously been reported. Phylogenetic analysis of the *gltA* gene for a subset of PCR-positive blood samples revealed sequences that were related to *Bartonella* described from vampire bats from Mexico, other Neotropical bat species, and streblid bat flies. Sequences associated with vampire bats clustered significantly by country but commonly spanned Central and South America, implying limited spatial structure. Stable and nonzero *Bartonella* prevalence between years supported endemic transmission in all sites. The odds of *Bartonella* infection for individual bats was unrelated to the intensity of bat flies ectoparasitism, but nearly all infected bats were infested, which precluded conclusive assessment of support for vector-borne transmission. While metagenomic sequencing found no strong evidence of *Bartonella* DNA in pooled bat saliva and fecal samples, we detected PCR positivity in individual saliva and feces, suggesting the potential for bacterial transmission through both direct contact (i.e., biting) and environmental (i.e., fecal) exposures. Further investigating the relative contributions of direct contact, environmental, and vector-borne transmission for bat *Bartonella* is an important next step to predict infection dynamics within bats and the risks of human and livestock exposures.

## Author summary

*Bartonella* are globally distributed bacteria that can cause endocarditis in humans and domestic animals. Bats have been implicated as a likely reservoir host for these bacteria, but little is known about how prevalence varies over time, routes of transmission, and the genetic diversity of *Bartonella* in bats. We present results from a two-year, spatially replicated study of common vampire bats, which have previously shown high infection prevalence of *Bartonella* and could pose risks of cross-species transmission due to their diet of mammal blood. We found that vampire bat *Bartonella* is genetically diverse, geographically widespread and endemic, and that individual-level infection risk is highest for large, male, non-reproductive bats. Phylogenetic analysis supported vector-borne transmission, and we found support for potential transmission through direct contact and fecal exposures through PCR. Confirming whether arthropod vectors are the main route of transmission for bat *Bartonella* is needed for understanding infection dynamics in bats and for predicting risks of cross-species transmission to humans and livestock.

## Introduction

Bats (Order: Chiroptera) serve as reservoir hosts for viruses of concern for human and animal health [1,2] including SARS coronavirus, rabies virus, filoviruses, and henipaviruses [3–6]. Bats can also harbor protozoa and bacteria of potential zoonotic relevance [7–9]. *Bartonella* spp. are of particular interest, as these Gram-negative bacteria cause bacteremia and endocarditis in both humans and livestock [10,11] and exhibit high genetic diversity in bats across multiple continents and species [12–17]. Moreover, phylogenetic analyses show bats are reservoirs of zoonotic *Candidatus* B. mayotimonensis [18–20], a causative agent of human endocarditis [21].

Given the zoonotic potential of bat-associated *Bartonella*, understanding transmission within bats is critical for understanding how *Bartonella* persists in bat populations and for assessing spillover risks [22,23]. Ectoparasites are frequently invoked as a transmission route [12,19,24], in part because vector-borne transmission occurs in other taxa [25,26] and because *Bartonella* has been identified in bat flies and ticks [27–29]. While some bat ticks can feed on humans [30], the high host specificity of bat flies [31,32] could limit opportunities for cross-species transmission through ectoparasites [31–33]. Transmission through close contact (e.g., biting) could occur given detection of *Bartonella* in dog and cat saliva [34,35] as well as human infection following scratches from dogs and cats [36]. Phylogenetic patterns of weak *Bartonella* host specificity in Neotropical bat communities could not only reflect transmission through close contacts between species in multi-species roosts, but could also stem from transmission through generalist vectors [15,24,37]. *Bartonella* might also be transmitted through exposure to feces between bats and to humans that enter roosts or to domestic animals exposed to bat feces [18,38].

In addition to the potential risks of cross-species transmission from bats to livestock and humans, the infection dynamics of *Bartonella* in bats are also uncertain. In rodents, *Bartonella* prevalence varies through time [39,40], but such patterns have not been well studied in bats [41]. Individual heterogeneities in infection by age and sex could also inform exposure patterns. Finally, global analyses suggest geographic structure in bat *Bartonella* genotypes, with notable differences in genotypes from Latin American and those from Africa, Europe, and Asia [42]. However, as such patterns appear driven by bat families restricted to different continents, analyses within narrower geographic and taxonomic ranges could inform the scale of *Bartonella* transmission and the role that dispersal plays in the spatial dynamics of this infection [43].

Common vampire bats (*Desmodus rotundus*) have high prevalence of *Bartonella* throughout their large geographic range in Latin America [15,16,24,44]. Vampire bats are of particular concern because they subsist on blood, which could create opportunities for *Bartonella* transmission to humans and livestock either from bites during blood feeding or through vector sharing facilitated by close proximity [45–48]. Here, we capitalize on a two-year, spatially replicated study of vampire bats to examine the genetic diversity and infection prevalence of *Bartonella*, including its geographic structure across the vampire bat range as well as individual and temporal correlates of infection status. To explore possible transmission routes of this bacterial pathogen, we also test for associations between bat fly infestation and *Bartonella* infection status, which would support vector-borne transmission, and by screening bat saliva and fecal samples for evidence of *Bartonella* DNA, which would support transmission through bites or grooming and environmental exposure to bacteria shed in feces, respectively.

## Materials and methods

### Vampire bat sampling

Samples were collected as described in Becker et al. [49] in 2015 and 2016 across seven sites in Peru (Departments of Amazonas [AM], Cajamarca [CA], and Loreto [LR]) and two sites in Belize (Orange Walk [OW] District). We sampled sites one to two times annually, capturing one to 17 individuals per site and sampling interval (S1 Table). To screen for *Bartonella* by PCR, up to 30 μL blood was stored on Whatman FTA cards at room temperature. To assess the presence of *Bartonella* in saliva and feces, we collected oral and rectal swabs from vampire bats in Peru. Swabs were preserved in 2 mL RNAlater (Invitrogen) at –80°C until laboratory analyses. For Peru sites sampled in 2016, we also recorded the number of bat flies per vampire bat [32].

### Ethics statement

Field procedures were approved by the University of Georgia Animal Care and Use Committee (A2014 04-016-Y3-A5) and the University of Glasgow School of Medical Veterinary and Life Sciences Research Ethics Committee (Ref08a/15); all procedures were conducted in accordance with accepted guidelines for humane wildlife research as outlined by the American Society of Mammalogists [50]. Bat capture, sample collection, and exportation were authorized by the Belize Forest Department under permits CD/60/3/15(21) and WL/1/1/16(17) and by the Peruvian Government under permits RD-009-2015-SERFOR-DGGSPFFS, RD-264-2015-SERFOR-DGGSPFFS, and RD-142-2015-SERFOR-DGGSPFFS. Access to genetic resources from Peru was granted under permit RD-054-2016-SERFOR-DGGSPFFS.

### Sequencing and phylogenetic analysis of vampire bat *Bartonella*

We analyzed samples that were previously screened for the presence of *Bartonella* by Becker et al. [49] using nested PCR to amplify a region of the citrate synthase gene (*gltA*) [51]. Among the *Bartonella*-positive samples, we randomly selected 5–10 positive samples per site for Sanger sequencing (*n* = 51). PCR products were purified with DNA Clean & Concentrator Kits (Zymo Research) and sequenced in both directions at the Georgia Genomics Facility. Resulting chromatograms were checked for quality and trimmed using Geneious (Biomatters) [52]. Post-trimmed forward and reverse sequences were assembled to create 348 base pair (bp) consensus sequences for each sample (*n* = 35; the quality of 16 chromatograms was too low). Sequences were considered part of the same genotype if they had >96% identity in *gltA*, an established cut-off for *Bartonella* species identification [53]. Sequences with >99.7% similarity were considered the same genetic variant [54]. We used a Chi-squared test with the *p* value generated via a Monte Carlo procedure with 1000 simulations [55] to assess whether our defined *Bartonella* genotypes were associated with region (i.e., Belize, eastern Peruvian Amazon, western Peruvian Amazon).

Two datasets were created for phylogenetic analyses. Dataset 1 was designed to assess the spatial structure of vampire bat–associated *Bartonella* across Latin America and therefore included our new sequences plus all previously reported *gltA* sequences from *Desmodus rotundus*. Dataset 2 was designed to capture the relatedness of the new sequences to all previously described *Bartonella* spp. regardless of isolation source, which comprised sequences generated in this study plus sequences obtained by conducting a BLAST search of each new sequence against GenBank, selecting the top 10 hits, and removing duplicates. For both datasets, consensus sequences were aligned using MAFFT. Phylogenetic analyses were carried out in MrBayes using the GTR+gamma model suggested by jModeltest2 [56]. For dataset 1 (*Desmodus*-associated sequences), we fit a codon partitioned substitution model by linking rates in codon positions 1 and 2 separately from codon position 3. For dataset 2, we used a simpler non-partitioned model because the more complex codon-partitioned model failed to converge. Dataset 2 included one sequence from *Brucella abortus* (Genbank Locus: MIJI01000003.1) as an outgroup [13]. Both datasets were run for 2.5 million generations with convergence checked and burn-in periods selected by assessing posterior traces in Tracer [57]. With dataset 1, we analyzed spatial clustering of vampire bat *Bartonella* by country (Belize, Guatemala, Mexico, Peru) using Bayesian Tip Association Significance Testing (BaTS) [58]. We here selected 1,000 trees from the posterior distribution of the MrBayes run and compared the country-level clustering to a null distribution from 10,000 trees with swapped tip associations [58].

### Statistical analyses of *Bartonella* infection status

We analyzed 193 samples from *Desmodus rotundus* to test whether temporal variation (season and year) and individual risk factors (e.g., age, sex) explain differences in *Bartonella* infection, using generalized mixed effects models (GLMMs) with binomial errors and a logit link fit with the *lme4* package in R [59,60]. We fit a single GLMM with an interaction between site and year to first test if prevalence varied over years across sampling locations; we excluded one site from this analysis (i.e., LR6) owing to sampling in only 2015. We included bat identification number (ID) as a random effect to account for multiple sampling of a small number of bats (*n* = 6). To assess seasonality in infection, we fit a separate GLMM with season (spring, summer, fall) as a predictor to data from two sites in Peru (AM1 and CA1) sampled across seasons (*n* = 63). We also fit a generalized additive model (GAM) with restricted maximum likelihood, binomial response, and a cyclic cubic regression spline for Julian date using the *mgcv* package [61]. We randomly selected repeatedly sampled bats, as including bat ID as a random effect here overfit the GAM.

To identify individual risk factors for *Bartonella* infection, we fit a single GLMM with bat age, forearm size, sex, and reproductive status; we also included interactions between sex and reproduction, sex and age, sex and forearm size, and reproduction and forearm size. We included categorical livestock biomass as a predictor in the GLMM to control for a previously observed negative association with *Bartonella* infection (121/173 positive bats) [49]. We fit this GLMM to a reduced dataset free of missing values (*n* = 189), included bat ID nested within site as a random effect, and calculated marginal *R*^*2*^ (*R*^*2*^_*m*_) to assess model fit [62]. Finally, for a data subset (*n* = 40 bats sampled in Peru in 2016), we fit two separate GLMs with bat fly intensity and presence as predictors to test whether ectoparasites explained *Bartonella* infection status. We fit a separate GLM with quasi-Poisson errors to test for sex and age differences in bat fly intensity.

### Assessment of *Bartonella* in saliva and feces

To examine possible transmission of *Bartonella* through biting, grooming, blood sharing, or fecal–oral exposure, we used metagenomic data from a parallel study to screen vampire bat saliva and fecal samples for *Bartonella* DNA. Three saliva and three fecal pools were shotgun sequenced, each containing nucleic acid extractions from swabs collected from ten vampire bats from one to two colonies. Pooled samples contained individuals from the same colonies of bats tested for *Bartonella* in blood through PCR, though not necessarily the same individuals.

As described previously [8], total nucleic acid was extracted from swabs and pooled equally according to RNA concentration. Pooled samples were DNAse treated and ribosomal RNA depleted, then cDNA synthesis was performed. Libraries were prepared using a KAPA DNA Library Preparation Kit for Illumina (KAPA Biosystems) modified for low input samples, and were individually barcoded during the PCR reamplification step [10]. The libraries included in this study were combined in equimolar ratios with other metagenomic libraries for sequencing on an Illumina NextSeq500 at the University of Glasgow Centre for Virus Research.

Reads were demultiplexed according to barcode and quality filtered using TrimGalore [63,64] with a quality threshold of 25, minimum read length of 75 bp, and clipping the first 14 bp of the read. Low complexity reads were filtered out using the DUST method and PCR duplicates removed using PRINSEQ [65]. We screened cleaned reads for the *Bartonella* genotypes detected in this study using nucleotide BLAST [66] against a custom database composed of the PCR-generated *Bartonella* sequences from this study, retaining only the best alignment (the high-scoring segment pair with the lowest e-value) for a single query–subject pair. To investigate the presence of *Bartonella* species other than genotypes detected in blood samples from vampire bats, cleaned reads were *de novo* assembled into contigs using the assembly only function of SPAdes [67]. Individual reads and contigs were screened for sequences matching *Bartonella* using protein alignment in Diamond [68], and close matches at the protein level were further characterized by nucleotide BLAST against the Genbank nt database. As the *gltA* gene is not highly transcribed, we also tested sequences for matches to *Bartonella* DNA-directed RNA polymerase subunit B (*rpoB*). We selected two *rpoB* sequences (Genbank accessions KY629892 and KY629911) from a study of vampire bat *Bartonella* [16] for which the same individuals exhibited 100% identity in the *gltA* gene to our blood sequences, and we used Bowtie2 to map quality filtered reads and contigs to those sequences [69].

Lastly, because nucleic acid pools were DNase treated for metagenomic sequencing, potentially reducing detection sensitivity, we used the same nested PCR protocol as used for blood-derived DNA [51] to test for the presence of *gltA* in DNA from individual saliva and fecal swab samples that made up metagenomic pools (*n* = 58; 28 saliva and 30 feces). As with our blood samples, we randomly selected a subset of positive amplicons for Sanger sequencing.

## Results

### Genetic diversity of vampire bat *Bartonella*

**Fig 1:**
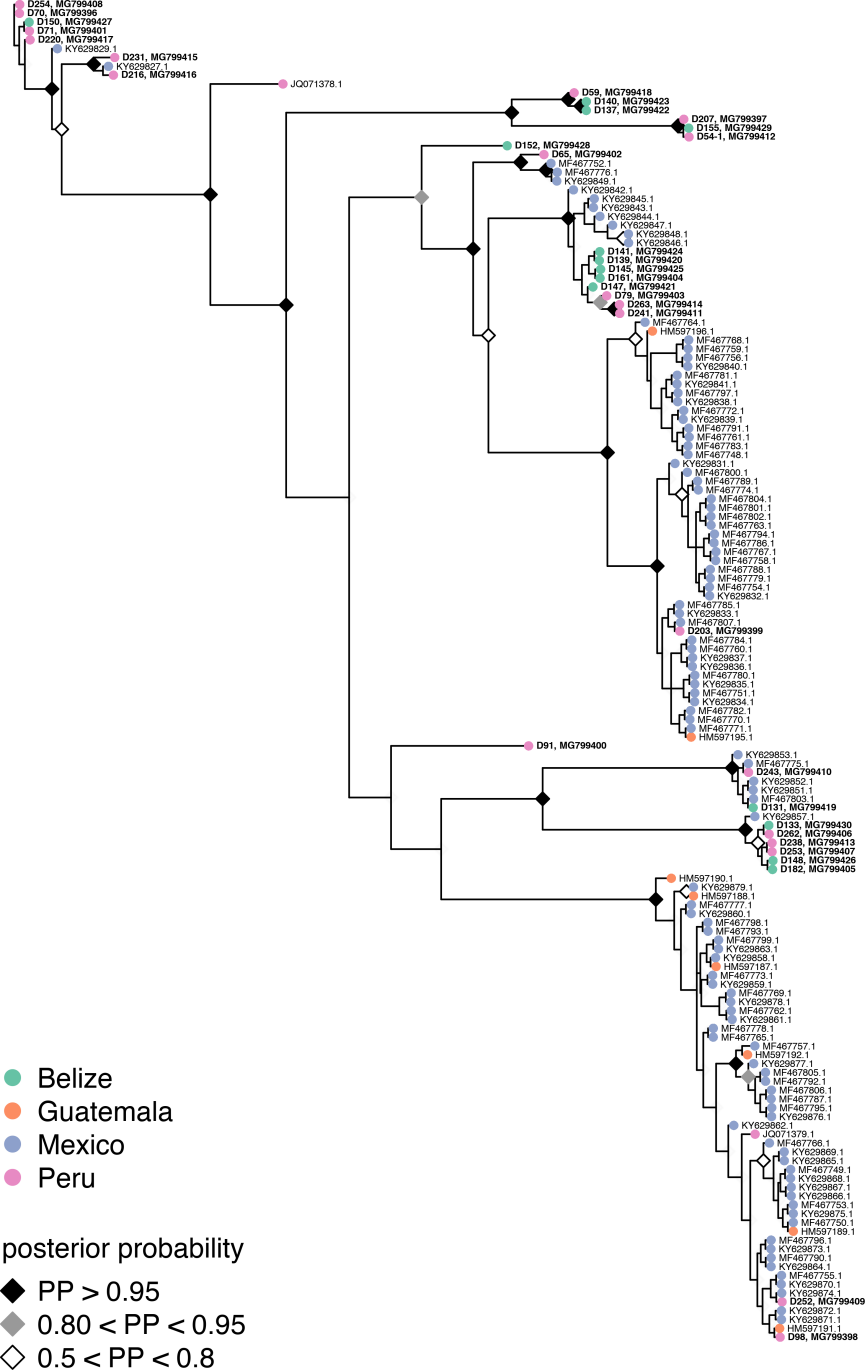
Phylogenetic relationships for the *gltA* gene among the sample of *Bartonella* genotypes detected in vampire bats from this study and vampire bat *Bartonella* genotypes from GenBank. All sequences are displayed with their GenBank accession numbers, and sequences from this study are listed in bold with bat ID numbers. The tips of all sequences are colored by geography, and diamonds depict posterior probabilities of nodes greater than 50%.

**Fig 2:**
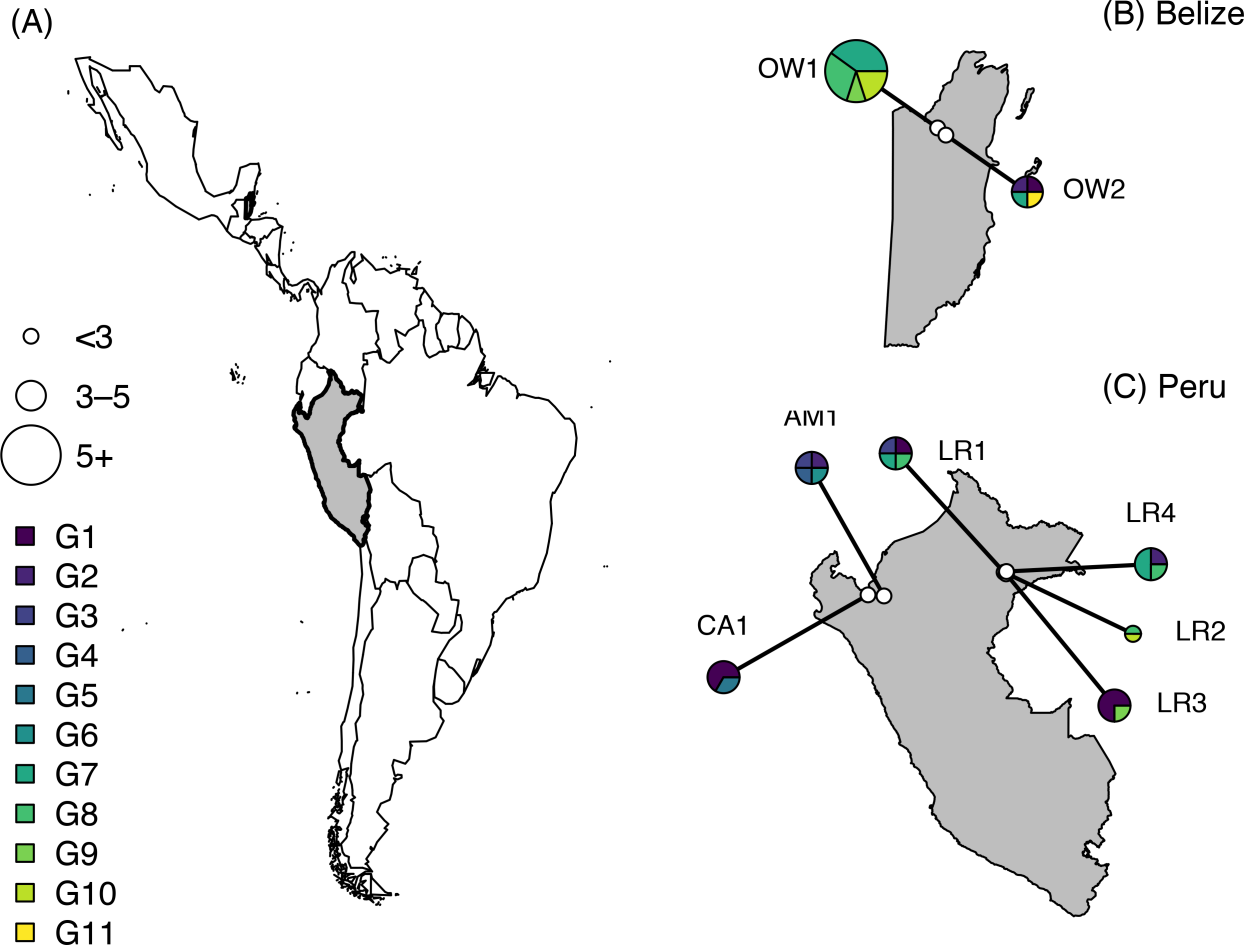
Distribution of the 11 paraphyletic genotypes identified from the 35 vampire bat *Bartonella* spp. *gltA* sequences. Belize and Peru are shown in grey with black outlines (A). Insets show the location of sampling sites (white), and pie graphs illustrate the genotype composition of *Bartonella* spp. per site (B and C) pooled across both study years. Pie charts are scaled by sample size. Shapefiles were obtained from the *maps* and *mapdata* packages in R [86].

*Bartonella* prevalence across the 193 vampire bats included in this study was 67%. Our phylogenetic analysis of 35 vampire bat *Bartonella* sequences showed 78.8–100% pairwise identity in *gltA* and revealed at least 11 paraphyletic genotypes (S2 Table). BaTS analysis of all *Desmodus*-associated *Bartonella* showed significant phylogenetic clustering by country (association index = 3.81, parsimony score = 31.51, *p*<0.001), although most vampire bat *Bartonella* genotypes were still widely distributed (Fig 1). For the 11 genotypes delineated from our 35 sequences, we observed no association with the geographic study region (*χ*^*2*^ = 23.3, *p* = 0.27). Genotypes 1 and 2 were detected across all regions, and genotypes 7–10 were detected within both Belize and Peru, highlighting the broad distribution of vampire bat *Bartonella* genotypes (Fig 2); however, genotype 3 was unique to both regions of Peru, genotypes 4–6 were unique to the western Peruvian Amazon, and genotype 11 was only detected in Belize.

**Fig 3:**
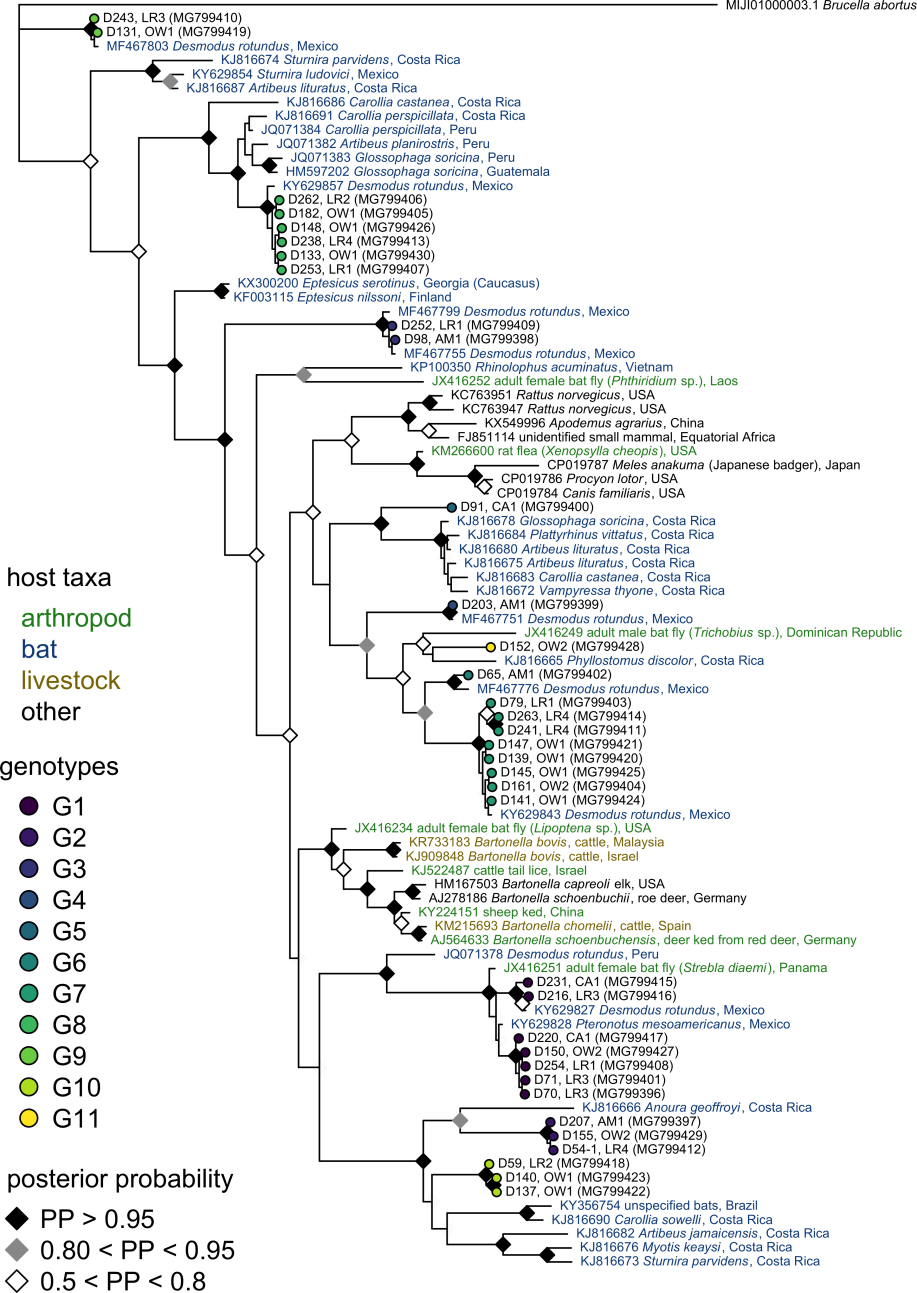
Phylogenetic relationships for the *gltA* gene among the sample of *Bartonella* sequences detected in vampire bats and top BLAST hits from GenBank (S3 Table). *Bartonella* sequences from this study are displayed with genotype, bat ID numbers, and accession numbers. Sequences from GenBank are colored by host taxa and provided with accession numbers, species, and sampling location. Diamonds depict posterior probabilities of nodes greater than 50%.

We also assessed the phylogenetic position of our vampire bat *Bartonella* sequences among known *Bartonella* genotypes (Fig 3, S3 Table). Half of our Peruvian and Belizian sequences (18/35) were nearly identical (>99.7% identity) to *Bartonella* from common vampire bats (*Desmodus rotundus*) from Mexico (e.g., GenBank accession numbers KY629837 and MF467803), again confirming the wide geographic distribution of these genotypes. Other sequences(9/35) fell within the same clade (>96% pairwise identity) as *Bartonella* from bat flies (*Strebla diaemi*) in Panama (JX416251), from Parnell's mustached bat (*Pteronotus parnellii*) in Mexico (e.g., KY629828), from phytophagous bats in Peru (e.g., *Carollia perspicillata*; JQ071384) and Guatemala (e.g., *Glossophaga soricina*; HM597202), or from Mexican vampire bats as noted above. Eight sequences were novel (<96% identity to GenBank sequences) but were most similar to *Bartonella* from phytophagous bats in Costa Rica (e.g., 90–93% to KJ816666 [*Anoura geoffroyi*]) and from Mexican vampire bats (e.g., 93% to MF467776).

Other novel sequences were weakly related to *B*. *bovis* from livestock in Israel and Malaysia (e.g., 89–90% to KJ909844 and KR733183), to *B*. *chomelii* from cattle in Spain (e.g., 89% to KM215693), to *B*. *capreoli* from elk in the United States (e.g., 89% to HM167503), and to *B*. *schoenbuchensis* from roe deer in Germany (e.g., 89% to AJ278186); indeed, posterior support for a bat–ruminant clade was low (<50%; Fig 3). Our BLAST procedure also identified weakly related *Bartonella* from rodents (e.g., 90% to *Rattus norvegicus* from the United States [KC763951] and 92% to *Apodemus agrarius* from China [KX549996]) and from carnivores (e.g., 89% to *Procyon lotor* from the United States [CP019786]). However, these livestock, rodent, and carnivore sequences formed separate phylogenetic clades from bat- and bat fly–derived *Bartonella* sequences (Fig 3). Despite the geographic proximity of our field sites to Brazil, our BLAST procedure found no *Bartonella* seqeunces similar to those recently described in Brazilian bat or rodent species [70–72]. An additional phylogenetic tree that includes these recently identified *Bartonella* is provided in S1 Fig.

### Temporal patterns in infection

**Fig 4:**
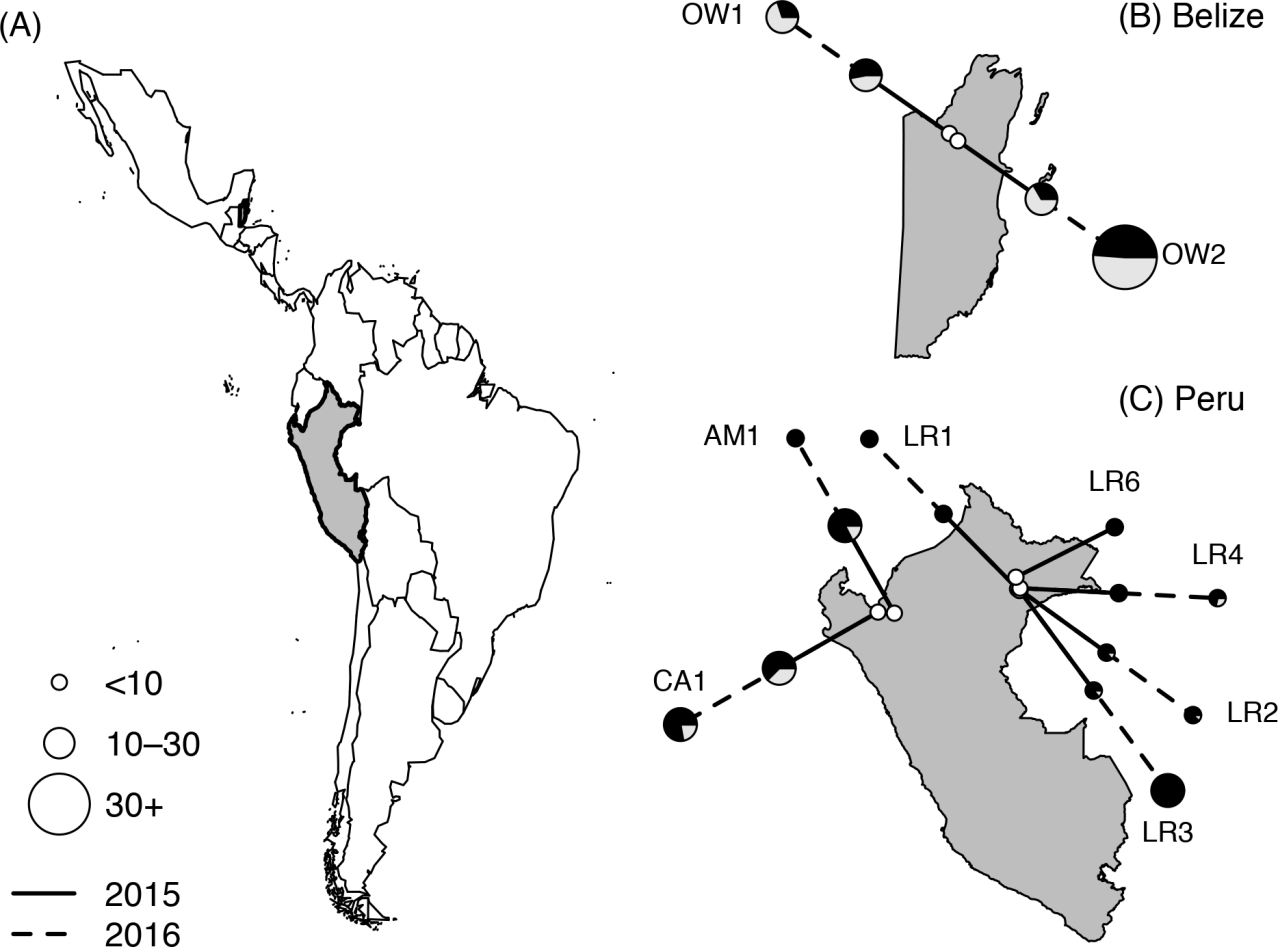
Vampire bat sampling sites in Latin America, with Belize and Peru shown in grey with black outlines (A). Insets show the location of sampling sites (white) and the prevalence of *Bartonella* per site (B and C) across study years (solid line = 2015, dashed line = 2016), with black denoting the proportion of infected bats. Pie graphs are scaled by sample size. Shapefiles were obtained from the *maps* and *mapdata* packages in R [86].

*Bartonella* was detected by PCR in all nine sites in each year, with prevalence ranging from 30–100% (Fig 4). Prevalence did not differ by year across all sites (*χ*^*2*^ = 3.13, *p* = 0.54) nor within individual sites (site*year; *χ*^*2*^ = 2.82, *p* = 0.90). The seasonality GLMM for the western Peruvian Amazon (*n* = 63) showed no difference in odds of infection between spring, summer, and fall (*χ*^*2*^ = 1.99, *p* = 0.37; S2 Fig). The GAM also showed no significant seasonal variation (*χ*^*2*^ = 0, *p* = 0.68; S2 Fig). Recaptures were rare (*n* = 6) but showed changes in infection from negative to positive (*n* = 2, 68–424 days) and from positive to negative (*n* = 2, 15–369 days; S3 Fig).

### Individual risk factors for infection

**Fig 5:**
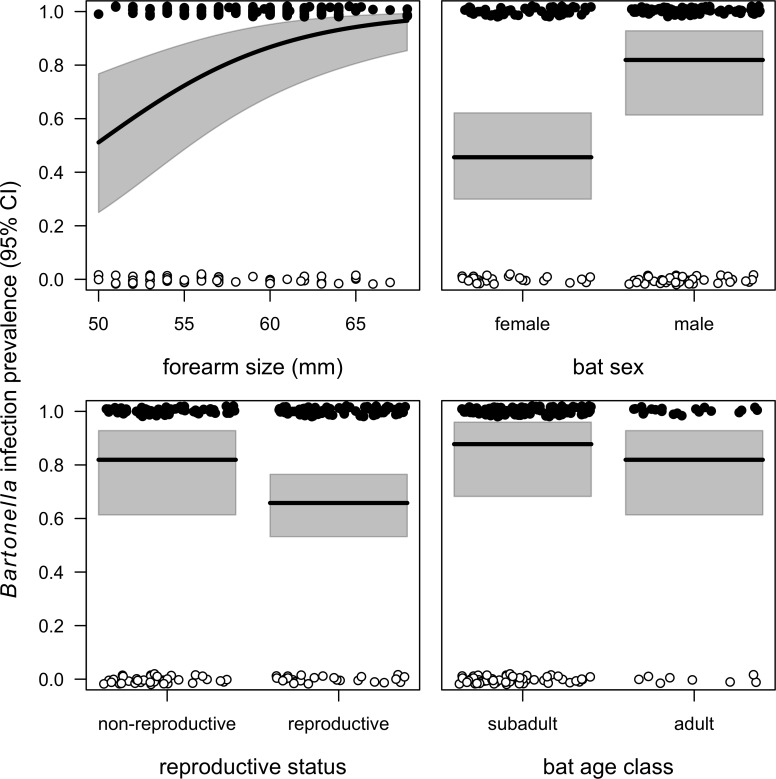
Modeled *Bartonella* infection prevalence (black line) and 95% confidence intervals (grey) from the GLMM of individual risk factors, displayed in order of effect size: forearm size (*χ*^*2*^ = 14.01), bat sex (*χ*^*2*^ = 8.48), reproductive status (*χ*^*2*^ = 2.79), and age (*χ*^*2*^ = 0.76). Individual data points are jittered and colored by infection status (black = positive, white = negative).

After controlling for site-level livestock biomass, vampire bat sex and forearm size were the strongest predictors of infection (Fig 5); no interactions were significant (all *χ*^*2*^*≤*1.18, *p*≥0.28) and were dropped from the final GLMM (*R*^*2*^_*m*_ = 0.28). The odds of *Bartonella* infection were highest for vampire bats with larger forearms (OR = 1.2, *p*<0.001) and for males (OR = 5.41, *p*<0.01), were marginally higher for non-reproductive individuals (OR = 2.36, *p* = 0.10), and did not differ between subadult and adult bats (OR = 1.58, *p* = 0.38); our sample did not contain juveniles. Individual bat fly intensities were highly variable (0–28, median = 7.5) and showed overdispersion (ϕ = 5.08 in an intercept-only quasi-Poisson GLM). The bat fly GLMs showed that neither ectoparasite intensity (OR = 0.98, *p* = 0.81) nor ectoparasite presence (*χ*^*2*^ = 1.13, *p* = 0.29) were associated with *Bartonella* infection status. We note that the majority of infected bats in this sample were infested with at least one bat fly (31/36), limiting conclusive assessment of the ectoparasite–infection relationship. Our multivariable quasi-Poisson GLM showed that ectoparasite load did not vary by bat sex (*χ*^*2*^ = 0.86, *p* = 0.35) or bat age (*χ*^*2*^ = 0.09, *p* = 0.77).

### Comparison of *Bartonella* detected in blood, saliva, and feces

There were no matches in any of the screened saliva and fecal metagenomic pools to the *Bartonella gltA* sequences detected in the blood or to previously published *Bartonella rpoB* sequences. The saliva pool from Amazonas had no matching *Bartonella-*like reads or contigs (S4 Table), while one read each from the Loreto and Cajamarca saliva pool was assigned as *Bartonella* by nucleotide BLAST; however, these reported species assignments should be interpreted cautiously as they are based on one read and percent identity was low. Pooled fecal samples from all departments of Peru contained *Bartonella*-like reads and contigs. *Bartonella ancashensis*, *B*. *australis*, and *B*. *bacilliformis* were all identified at both the read and contig level in fecal samples. However, because percent identity was relatively low, species assignments should again be interpreted cautiously. Subsequent BLAST hits following the top hit also frequently (though not always) matched to *Bartonella*, suggesting the presence of poorly characterized *Bartonella* species present or that these may be matches to other bacteria.

**Fig 6:**
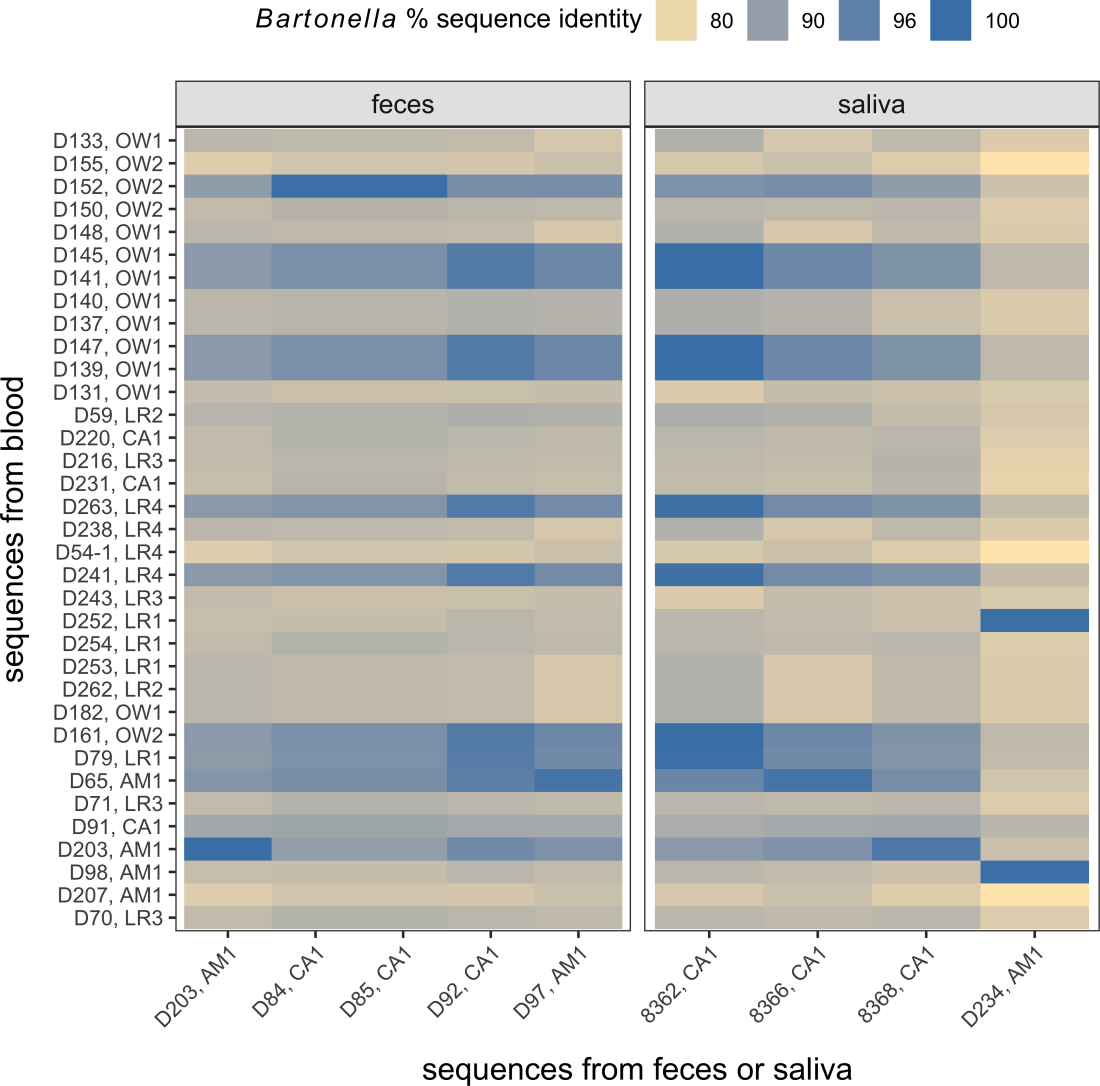
Heatmap of pairwise similarity (%) between the 35 vampire bat–associated *Bartonella* sequences from this study and the nine *Bartonella* sequences derived from a subset of positive saliva and fecal samples, calculated with Kimura's 2-parameters distance model. Sequences with greater than 96% similarity were assumed to be part of the same *Bartonella* genotype.

In contrast, nested PCR of individual swabs detected *gltA* in 21.4% of saliva samples (6/28) and 30% of fecal samples (9/30). For swab samples that were also assessed by PCR in blood (*n* = 15 for saliva, *n* = 28 for feces), both corresponding positive saliva samples were positive in blood; most positive fecal samples were also PCR positive in blood, although one fecal-positive sample was PCR negative in blood (S4 Fig). For our random subset of sequenced positive saliva (*n* = 4) and fecal (*n* = 5) samples, phylogenetic analyses suggested that all sequences shared a minimum of 97% identity to one or more of our 35 blood-derived *Bartonella* sequences (S5 Table, Fig 6). In many cases, saliva and fecal sequences were the same genotype as blood sequences derived from the same geographic region (e.g., the saliva sequence from D234 shared >96% identity to the blood sequence from D98, both from AM1). For the one case in which we sequenced positive samples from the same individual bat (i.e., D203), both the blood sequence and fecal sequence shared 100% identity (S5 Table, Fig 6). For the few sequences at the lower range of our similarity spectrum, BLAST still demonstrated that the closest relatives were all derived from vampire bats (i.e., 8368 from CA1 was identical to MF467797 from Mexico).

## Discussion

Despite an increasing focus on *Bartonella* genetic diversity and prevalence in bat communities, individual risk factors and transmission routes of this pathogen in bats remain largely unknown. For example, a survey of vampire bats within Guatemala found neither geographic, dietary, demographic, or viral coinfection correlates of *Bartonella* infection status [44]. Using a larger sample across a more diverse range of study sites and timepoints, we here show that *Bartonella* is genetically diverse, geographically widespread and endemic within vampire bat populations, and that individual-level odds of infection are highest for large, male, and non-reproductive bats. Furthermore, we use several approaches to suggest vector-borne transmission to be likely in addition to possible direct contact and environmental sources of *Bartonella* exposure in bats.

The *Bartonella* genotypes we identified were paraphyletic and closely related to those from other vampire bat populations, other Neotropical bat species, or bat flies. Although BLAST also identified *Bartonella spp*. sequenced from rodents, carnivores, and livestock within our hit selection criteria, these consistently formed separate phylogenetic clades that did not contain bat- or bat fly–derived *Bartonella* (Fig 3). These phylogenetic patterns indicate that *Bartonella* has commonly shifted between bat host species in the Americas but do not support frequent transmission between bats and other host groups. Our BaTS analysis also showed that vampire bat *Bartonella* sequences clustered by country more than expected by chance. However, given that several *Bartonella* genotypes were present in vampire bats from both Central and South America, we suspect this clustering mostly resulted from variation in locally abundant genotypes rather than true barriers to the spread of *Bartonella*. Because vampire bats are largely sedentary and non-migratory [45], dispersal of these *Bartonella* genotypes across large distances is unlikely to be attributable to bat movement alone. *Bartonella* genotypes may also have infected vampire bats over long evolutionary timescales, and thus the biogeography of the pathogen may have followed that of its host. Alternatively, *Bartonella* dispersal by other arthropod vectors (e.g., ticks) or other bat species that share *Bartonella* genotypes with vampire bats may be conceivable and could be resolved by further field surveys combined with population genetic analyses of alternative bat host species, arthropod vector species, and *Bartonella* genotypes.

Few studies have examined temporal patterns of bat *Bartonella*, emphasizing the general need for more longitudinal studies to understand how pathogens persist in bat populations [1,6]. Here, *Bartonella* was detected at relatively high prevalence across both study years within each sampling site, and neither year nor its interaction with site were predictive within our analyses. Similarly, no temporal patterns in *Bartonella* were observed for a limited sample of *Myotis mystacinus*, *Pipistrellus* spp., *Myotis daubentonii*, and *Nyctalus noctula* in the United Kingdom [41]. Such findings contrast with highly seasonal *Bartonella* infections in rodents, which show high prevalence in summer and fall due to seasonality in birth and ectoparasite intensity [39,40]. The lack of seasonality in our western Peruvian Amazon sample in particular could simply be due to low statistical power; alternatively, no seasonality in infection could also be explained by the non-seasonal or less-pronounced birth pulses observed for vampire bats (but see [73]). While high *Bartonella* prevalence in bats has been proposed to stem from persistent infection [15], this seems unlikely, as we observed possible clearance of infection in some recaptured bats. While this could also reflect bacteria DNA loads too low to be detected by PCR, infection risk did not increase with age, as would also be expected if bats could not clear infection [74]. However, we do note that our sample only contained adult (*n* = 162) and subadult (*n* = 28) bats, limiting more robust tests of age-dependent infection. Alternatively, *Bartonella* infections could be chronic and vary in infection intensity over time or could become latent (i.e., be undetectable in erythrocytes but persist in endothelial cells), particularly as infection does not appear to confer long-term immunity [36]. Such explanations could be confronted in future work with larger sample sizes of recaptured bats, multiple assessments of infection status over time, and quantitative PCR.

Bat forearm size, sex, and reproductive status were important predictors of *Bartonella* infection status, with odds of infection being higher in larger, male, and non-reproductive bats. While subadult status itself was not an important predictor of *Bartonella* infection, these findings could suggest higher risk in young male bats that are relatively large for their age. Our previous work has shown stronger innate immune defense (i.e., bacterial killing ability) in reproductive (mostly male) vampire bats, also suggesting greater susceptibility of non-reproductive hosts [49]. Similarly, subadults across a Mexican bat community also had higher odds of *Bartonella* infection [16], and young male vampire bats play key roles in the long-distance dispersal of rabies virus [43] and display higher rates of rabies exposure, possibly owing to more direct contacts during the first year of life [75]. Larger forearm size could also relate to direct contact if larger bats are more dominant and aggressive, as found in other phyllostomid bat species [76].

Although vector-borne transmission is generally assumed for *Bartonella* in other hosts [12,19,25,77], including some Neotropical bats [16,78], infection status in vampire bats was not associated with bat fly intensity. Further supporting this observation, male bats had higher odds of *Bartonella* infection but did not differ in their bat fly intensities compared to females. Weak correspondence between bat fly intensity at the time of sampling and *Bartonella* infection thus may cast doubt on bat flies as a primary transmission route. Time lags could provide one reason for this discrepancy, given that new *Bartonella* infections may take days or weeks to develop and become detectable and over which time ectoparasite load may have changed due to the mobile nature of bat flies [26,36,79]. On the other hand, it is possible that vector presence (rather than abundance) is a more important driver of transmission. Unfortunately, nearly all bats in this study had ectoparasites, so comparisons of *Bartonella* presence in bats with and without bat flies had little statistical power (31/36 *Bartonella*-positive bats were infested with at least one bat fly). Given that ectoparasitism predicted *Bartonella* infection more generally across a Mexian bat community [16], larger sample sizes with greater variation in bat fly intensity could provide better inference. However, our phylogenetic analysis does provide a tentative line of evidence supporting vector-borne transmission, as several of *Bartonella* genotypes fell within the same clade as *Bartonella* from streblid bat flies [29]. A recent survey of Mexican bats and their sympatric bat flies suggested that corresponding hosts and their bat flies had varied *Bartonella* genotypes, although one vampire bat did show complete sequence homology with the *Bartonella* from its paired bat flies [37]. As genetic similarity between *Bartonella* in bat flies and hosts has been interpreted as evidence of vector-borne transmission in other bat species [29,54], further assessments of *Bartonella* genotypes between vampire bats and their various ectoparasites (bat flies but also ticks) would shed additional light on possible routes of vector-borne transmission.

Lastly, we analyzed bat saliva and feces using metagenomics and PCR to explore alternative transmission routes, namely through close contact and fecal exposure. Metagenomics detected no *Bartonella* DNA matching to *gltA* or *rpoB* in either saliva or fecal pools. This absence could be explained in that the short sequences (345–425 bp) used as targets, and the large size of bacterial genomes together make the likelihood of detecting a specific gene low. However, the *Bartonella-*like reads and contigs recovered from saliva and feces were short fragments (51–258 bp) and showed low homology to known *Bartonella* from GenBank (S4 Table). Notably, we used a similar approach to search for other bacteria (i.e., hemoplasmas) and found clear evidence of their presence [8]. While this could suggest true absence of *Bartonella* from bat saliva and feces, our PCR found *Bartonella* in a subset of individual saliva and fecal samples. This discrepancy between methods could stem from treating saliva and fecal pools with DNase before metagenomic sequencing. Furthermore, phylogenetic analyses confirmed that these sequences were closely related to those identified in blood, which argues against these PCR positives only representing bacteria derived from environmental contamination or from feeding on prey. PCR results further showed strong correspondence between blood and saliva, suggesting that *Bartonella* infection may be systemic in vampire bats. While fecal and blood PCR results also mostly matched, we found one case where a bat was negative in blood but positive in feces. As consumption of ectoparasites during grooming has been observed in other bat families (e.g., Pteropodidae [80]), this discrepancy could suggest the incidental ingestion of ectoparasites during grooming and that this does not lead to systemic infection more generally indicated by the concordance between blood, saliva, and fecal positives and their close genetic similarity.

Similar prevalence of *Bartonella* in saliva and feces suggests that direct contact and environmental exposure could serve as complemenry transmission routes to arthropod vectors. The presence of *Bartonella* in saliva samples contrasts with previous work showing an absence of *Bartonella* in vampire bat saliva [44,81], providing evidence for possible direct transmission. *Bartonella* in fecal samples could also suggest environmental transmission between bats [18]. Both saliva-borne and fecal–oral transmission of vampire bat *Bartonella* could further pose potential risks to humans or livestock, either through bites during feeding or by environmental exposure of humans that enter roosts or to domestic animals exposed to bat feces [18,38,48,82]. For the former pathway, however, a recent survey of *Bartonella* in Mexican ruminants did not identify being bitten by vampire bats as a risk factor for infection [81], and our phylogenetic results provide relatively more support for the possibility of vector-borne transmission. Vector-borne transmission of vampire bat *Bartonella* might reduce their potential to infect humans or livestock, given the high host specificity of most bat flies [31,32]. However, ectoparasite transfer between individuals could still occur during pupal deposition and close contact [83], facilitating *Bartonella* transmission within vampire bat colonies and to other bat species. While our analyses of ectoparasitism only considered bat flies, we have observed heavy tick burdens of vampire bats in other field sites (e.g., Belize). *Bartonella* has been detected in ticks infesting other bats [28], and these ectoparasites could also be more likely to facilitate cross-species transmission [30]. Metagenomics also potentially identified *Bartonella ancashensis* and *B*. *bacilliformis* in vampire bat fecal samples, and these species cause notable infectious disease in humans likely through phlebotomine sand flies in Andean regions of Peru [84,85]. Controlled infection trials and more extensive phylogenetic analyses of *Bartonella* in vampire bats, their various ectoparasites, and sympatric prey are therefore needed to examine the contributions of different transmission routes for bacterial spread within vampire bats and to recipient prey and to confirm whether saliva and feces represent viable transmission routes. Given the high rates of bat bites and proximity to wildlife, humans, and domestic animals that define vampire bat ecology, such efforts to verify the possibility and frequency of oral and environmental exposures would elucidate *Bartonella* transmission dynamics in this common host species and the risks of cross-species transmission.

## Supporting information

S1 Table Sample size, sampling dates, and *Bartonella* prevalence in vampire bats. (CSV)

S2 Table Pairwise similarity (%) between the 35 vampire bat–associated *Bartonella* sequences from this study, calculated with Kimura's 2-parameters distance model. Sequences with greater than 96% similarity were assumed to be part of the same genotype for downstream analyses.
(CSV)

S3 Table Pairwise similarity (%) between each vampire bat *Bartonella* genotype from this study and the top 10 BLAST hits from GenBank. (CSV)

S4 Table *Bartonella*-like reads and contigs detected in pooled saliva and fecal samples using metagenomics. Department describes where samples in a pool were taken. *Bartonella*-like reads were identified by Diamond protein blast, and read matches are a list of those that were identified as *Bartonella* based on nucleotide blast. *Bartonella*-like contigs were identified by Diamond protein blast, and contig matches are a list of those that were identified as *Bartonella* based on nucleotide blast. Length is contig length in base pairs, and K-mer coverage is the depth of coverage for the largest k-value used in the SPAdes assembly.
(CSV)

S5 Table Pairwise similarity (%) between the 35 vampire bat–associated *Bartonella* sequences from this study and the nine sequences derived from a subset of positive saliva (*n* = 4) and fecal (*n* = 5) samples, calculated with Kimura's 2-parameters distance model. Sequences with greater than 96% similarity were assumed to be part of the same *Bartonella* genotype.
(CSV)

S1 Fig Phylogenetic relationships for the *gltA* gene among the sample of *Bartonella* sequences detected in vampire bats, top BLAST hits from GenBank (S3 Table), and sequences from recent studies of bat and rodent *Bartonella* from Brazil. *Bartonella* seqeunces from our study are listed with genotype, bat ID numbers, and accession numbers. Sequences from GenBank are colored by host taxa and provided with accession numbers, host species, and sampling location; red tips display recent *gltA* sequences from Brazilian bats and rodents.
(TIFF)

S2 Fig Lack of seasonality in vampire bat *Bartonella* from western Peruvian Amazon sites where colonies were sampled multiple times in each of the two years. Modeled prevalence (black line) and 95% confidence intervals (grey) from the GLMM (left; *n* = 63) and GAM (right; *n* = 61). Data points are jittered and colored by infection status (black = positive, white = negative).
(TIFF)

S3 Fig Dynamics of *Bartonella* infection status for recaptured vampire bats (*n* = 6) during 2015 and 2016. Infected bats are shown in red, uninfected bats are shown in black.
(TIFF)

S4 Fig Counts of positive and negative *Bartonella* PCR results for saliva (A) and fecal samples (B) for which blood was also assessed for evidence of infection.
(TIFF)
