# Supplementary figures and images for: Genetic diversity, infection prevalence, and possible transmission routes of Bartonella spp. in vampire bats
Source: PLoS Negl Trop Dis. 2018 Sep 27;12(9):e0006786. doi: 10.1371/journal.pntd.0006786 (PMC6159870; doi:10.1371/journal.pntd.0006786)

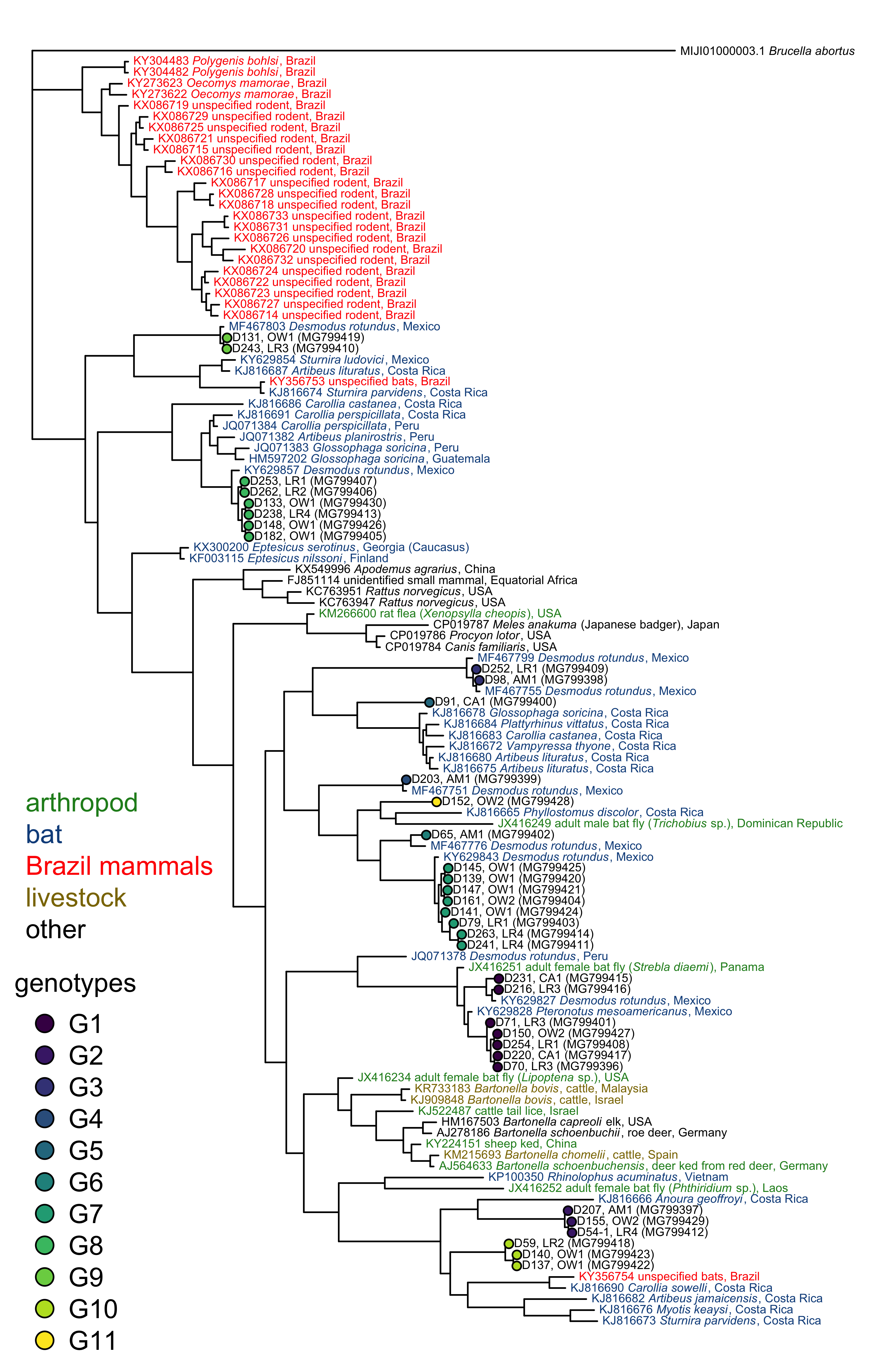

Supplement: S1 Fig — Bartonella seqeunces from our study are listed with genotype, bat ID numbers, and accession numbers. Sequences from GenBank are colored by host taxa and provided with accession numbers, host species, and sampling location; red tips display recent gltA sequences from Brazilian bats and rodents. (TIFF) [file pntd.0006786.s006.tiff]

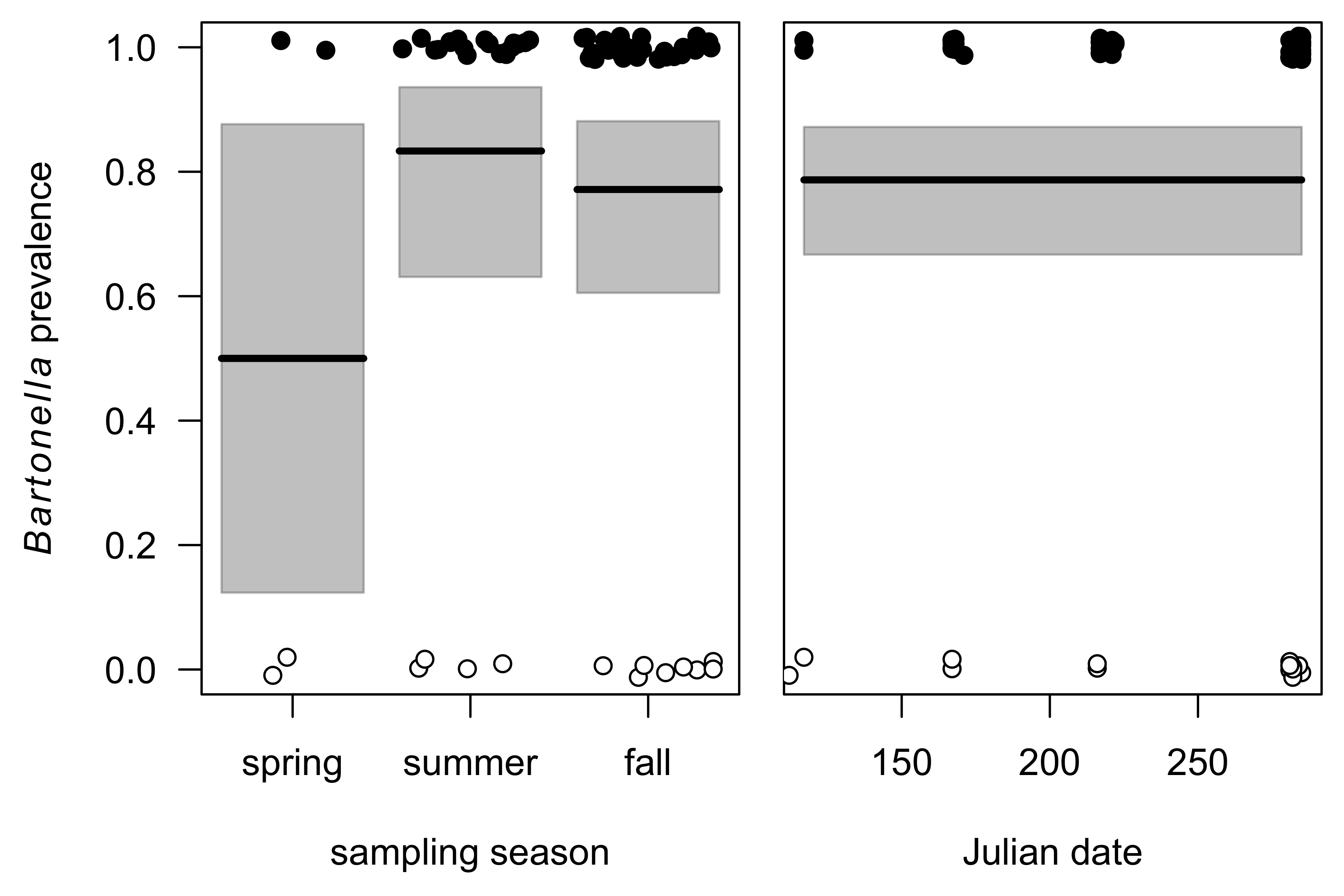

Supplement: S2 Fig — Modeled prevalence (black line) and 95% confidence intervals (grey) from the GLMM (left; n = 63) and GAM (right; n = 61). Data points are jittered and colored by infection status (black = positive, white = negative). (TIFF) [file pntd.0006786.s007.tiff]

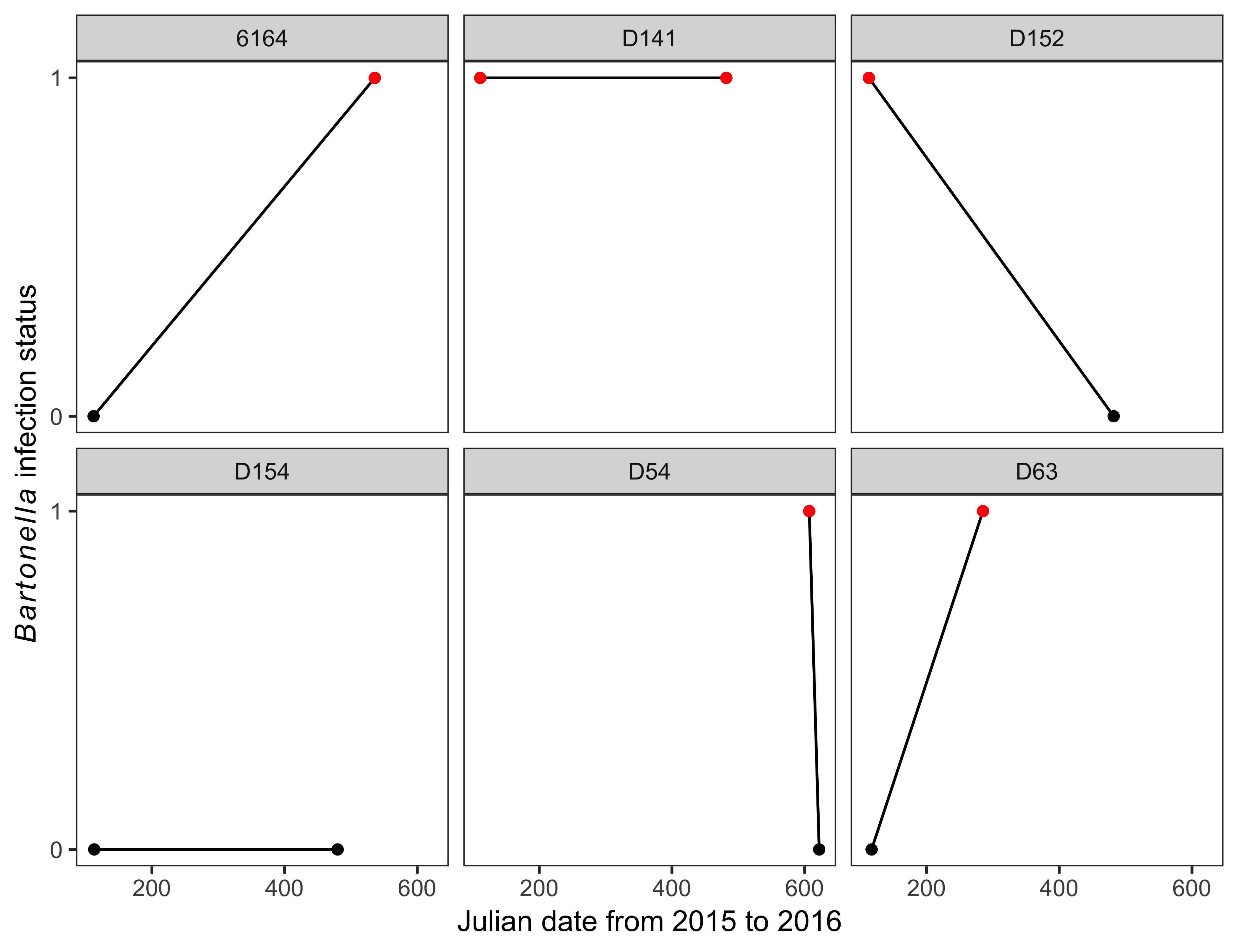

Supplement: S3 Fig — Infected bats are shown in red, uninfected bats are shown in black. (TIFF) [file pntd.0006786.s008.tiff]

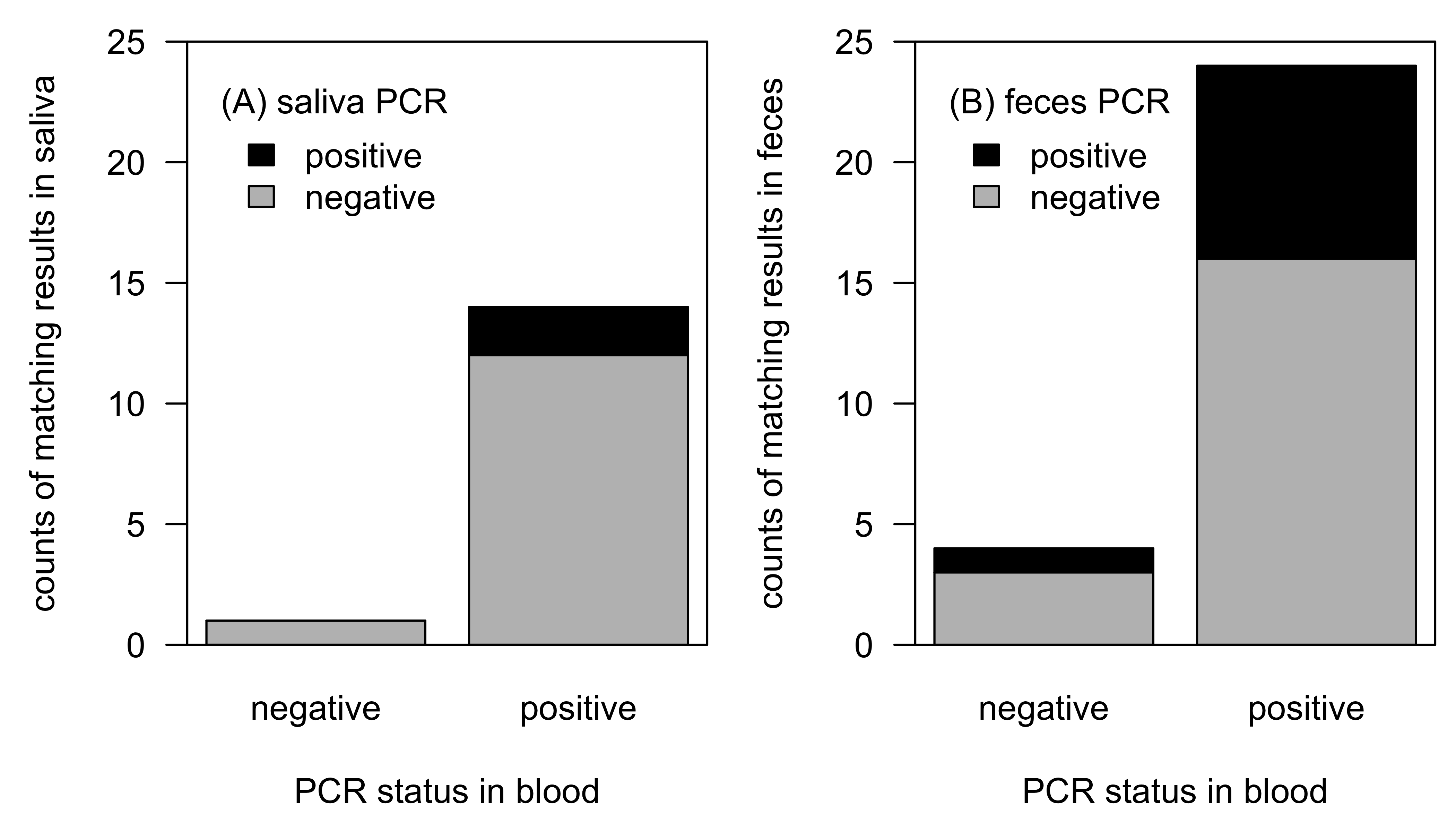

Supplement: S4 Fig — Counts of positive and negative Bartonella PCR results for saliva (A) and fecal samples (B) for which blood was also assessed for evidence of infection. (TIFF) [file pntd.0006786.s009.tiff]
